# Stereoselective Regulations of P-Glycoprotein by Ginsenoside Rh2 Epimers and the Potential Mechanisms From the View of Pharmacokinetics

**DOI:** 10.1371/journal.pone.0035768

**Published:** 2012-04-18

**Authors:** Jingwei Zhang, Fang Zhou, Fang Niu, Meng Lu, Xiaolan Wu, Jianguo Sun, Guangji Wang

**Affiliations:** Key Lab of Drug Metabolism and Pharmacokinetics, China Pharmaceutical University, Nanjing, Jiangsu, China; Johns Hopkins University, United States of America

## Abstract

Chirality is an interesting topic and it is meaningful to explore the interactions between chiral small molecules and stereoselective biomacromolecules, with pre-clinical and clinical significances. We have previously demonstrated that 20(S)-ginsenoside Rh2 is an effective P-glycoprotein (P-gp) inhibitor in *vitro* and in *vivo*. Considering the stereochemistry of ginsenoside Rh2, in our present study, the regulatory effects of 20(R)-Rh2 on P-gp were assayed *in vivo*, and the differential regulations of P-gp by ginsenoside Rh2 epimers *in vivo* were compared and studied. Results showed that 20(S)-Rh2 enhanced the oral absorption of digoxin in rats in a dose-dependent manner; 20(R)-Rh2 at low dosage increased the oral absorption of digoxin, but this effect diminished with elevated dosage of 20(R)-Rh2. Further studies indicated stereoselective pharmacokinetic profiles and intestinal biotransformations of Rh2 epimers. *In vitro* studies showed that Rh2 epimers and their corresponding deglycosylation metabolites protopanaxadiol (Ppd) epimers all exhibited stereoselective regulations of P-gp. In conclusion, in view of the *in vitro* and *in vivo* dispositions of Rh2 and the regulations of P-gp by Rh2 and Ppd, it is suggested that the P-gp regulatory effect of Rh2 *in vivo* actually is a double actions of both Rh2 and Ppd, and the net effect is determined by the relative balance between Rh2 and Ppd with the same configuration. Our study provides new evidence of the chiral characteristics of P-gp, and is helpful to elucidate the stereoselective P-gp regulation mechanisms of ginsenoside Rh2 epimers *in vivo* from a pharmacokinetic view.

## Introduction

Chirality is a quite common feature for both biomacromolecules and small-molecules in nature and in our daily life. Biomacromolecules have the potential to stereoselectively recognize and dispose the ligands. For example, it has been shown that S-verapamil is significantly different from R-verapamil in plasma protein binding and systemic clearance [1], [2]. On the other hand, small-molecules also stereoselectively take their biological actions. Taking propoxyphene as an example, dextropropoxyphene is an analgesic, whereas levopropoxyphene is an antitussive agent [3]. Warfarin is another example. At physiological concentrations, R-warfarin interacts with pregnane X receptor (PXR) and significantly induces CYP3A4 and CYP2C9 mRNAs, while S-warfarin does not show such effects [4]. As mentioned above, it is interesting and important to explore the interactions between chiral small molecules and stereoselective biomacromolecules, with pre-clinical and clinical significances.

Ginsenosides, the main effective constituents of ginseng, have a broad range of therapeutic applications. The basic structure of ginsenoside is tetracyclic triterpenoid, with many chiral carbones in the molecule. Particularly, the chirality of carbon-20 contributes to the two stereoisomers of each ginsenoside. They are called epimers. It is very likely that the two epimers of ginsenoside have different biological characteristics. 20(S)-ginsenoside Rg3 but not 20(R)-ginsenoside Rg3 inhibited the Ca^2+^, K^+^ and Na^+^ channel currents in a dose- and voltage-dependent manner [5], [6]. In human fecal microflora, the amount of 20(S)-ginsenoside Rg3 transforming to 20(S)-ginsenoside Rh2 was 19-fold higher than that of 20(R)-ginsenoside Rg3 transforming to 20(R)-ginsenoside Rh2 [7]. On the other hand, as the deglycosylation metabolite of Rg3, ginsenoside Rh2 also exhibited stereoselective activities. 20(S)-ginsenoside Rh2 but not 20(R)-ginsenoside Rh2 inhibited the proliferation of both androgen-dependent and –independent prostate cancer cells [8]. Interestingly, 20(R)-ginsenoside Rh2 is a selective osteoclastgenesis inhibitor without any cytotoxicity, while 20(S)-ginsenoside Rh2 showed weak osteoclastgenesis inhibition but had strong cytotoxicity in osteoclasts [9]. We have previously examined the pharmacokinetic profile of ginsenoside Rh2 and observed its poor bioavailability (absolute bioavailabilities were about 4.0–6.4% when 1–9 mg/kg Rh2 were i.g. administered to rats) [10]. We found that stereochemistry was one of the causes to poor oral absorption, because 20(S)-ginsenoside Rh2 and 20(R)-ginsenoside Rh2 exhibited different membrane permeabilities [11]. Hence, the stereochemistry of the hydroxyl group at carbon-20 plays an important role in the activities of ginsenoside epimers.

P-glycoprotein (P-gp), a member of drug transporters, mediates not only the transport of endogenous substances but also of the exogenous therapeutic drugs. As biomacromoleucles, P-gp owns the ability to distinguish the ligands stereoselectively, and contributes to different dispositions of the chiral ligands [12]. For example, P-gp ATPase hydrolysis and P-gp substrate recognition was stimulated by *cis*-flupentixol while inhibited by *trans*-flupentixol [13]. Recently, the structure of mouse P-gp, with 87% sequence identity to human P-gp, has been reported [14]. It was found that P-gp could distinguish between QZ59-RRR and QZ59-SSS, two stereoisomers of cyclic peptides, through different binding locations, orientation and stoichiometry with P-gp.

**Figure 1:**
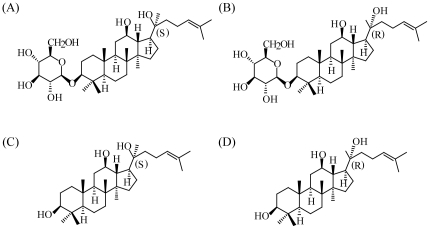
Chemical structures of ginsenosides. (A) 20(S)-Rh2, (B) 20(R)-Rh2, (C) 20(S)-Ppd and (D) 20(R)-Ppd.

It is very interesting to discuss the interactions between P-gp and chiral small molecules. However, the related reports are limited. Recently, we have demonstrated that 20(S)-ginsenoside Rh2 is an effective P-gp inhibitor both in *vitro* and in *vivo* [15]. Considering the stereochemistry of ginsenoside Rh2, in our present study, the regulatory effects of 20(R)-Rh2 (Fig. 1) on P-gp were assayed *in vivo*. For a comparative understanding of the differential regulation of P-gp by ginsenoside Rh2 epimers *in vivo*, the pharmacokinetics of Rh2 epimers *in vivo*, the possible metabolism, and evaluation of P-gp regulatory effects *in vitro* were all included. Moreover, the differential P-gp regulations of Rh2 epimers were further confirmed by applying Rh2 epimers as P-gp regulators in reversal of P-gp mediated multi-drug resistance. Our study provides a new case describing the chiral characteristics of P-gp. It is also a meaningful trial to elucidate the stereoselective P-gp regulation mechanisms of ginsenoside Rh2 epimers *in vivo* from a pharmacokinetic view.

## Results

### Effects of 20(S)-Rh2 and 20(R)-Rh2 on oral pharmacokinetics of digoxin in rats

**Figure 2:**
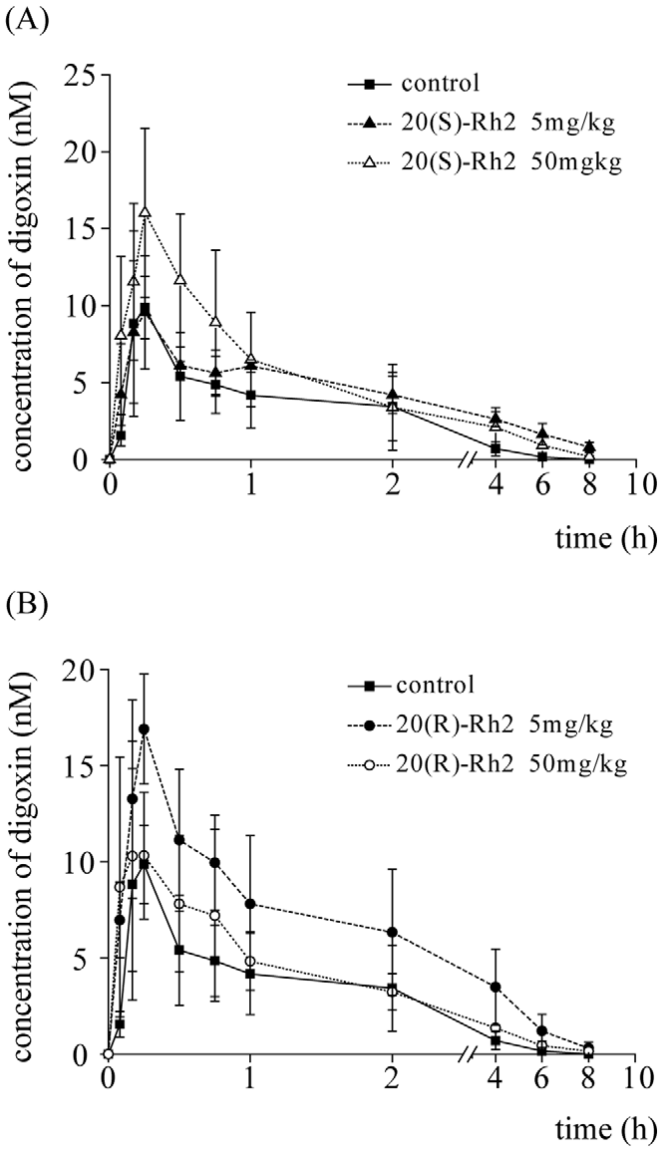
Plasma concentration-time curves of digoxin in 20(S)-Rh2 or 20(R)-Rh2 pre-treated male SD rats. The control groups were given an i.g. dose of 0.25 mg/kg digoxin. The pre-treated groups were administered an i.g. dose of 5 mg/kg and 50 mg/kg 20(S)-Rh2 (A) or 5 mg/kg and 50 mg/kg 20(R)-Rh2 (B), 2 h prior to digoxin dose. Data are expressed as mean ± S.E., n = 5 per group.

Digoxin has been proved as a classic P-gp substrate, and its intestinal absorption is mainly restricted by P-gp [16], [17]. When 20(S)-Rh2 was i.g. administered to rats prior to i.g. administration of digoxin, the oral absorption of digoxin was enhanced with increasing concentrations of 20(S)-Rh2 (Fig. 2A). The AUC and C_max_ of digoxin were elevated by 1.8-fold and 1.6-fold respectively by 50 mg/kg 20(S)-Rh2 (Table 1). However, it was different in the case of 20(R)-Rh2. When 20(R)-Rh2 was i.g. administered to rats at 5 mg/kg prior to i.g. administration of digoxin, the AUC and C_max_ of digoxin were significantly enhanced (Table 1). But, when the dosage of 20(R)-Rh2 was elevated to 50 mg/kg, the absorption of digoxin was not changed significantly compared with control group (Fig. 2B). The dose-effect trends of 20(S)-Rh2 and 20(R)-Rh2 on the oral pharmacokinetics of digoxin were just opposite.

**Table 1:** Pharmacokinetic parameters of digoxin after a single i.g. administration in the absence or presence of 20(S)-Rh2 or presence of 20(R)-Rh2 in rats.

Parameters	Digoxin (0.25 mg/kg i.g.)				
	Control	20(S)-Rh2		20(R)-Rh2	
		5 mg/kg	50 mg/kg	5 mg/kg	50 mg/kg
AUC _0–12_ (nmol/L×h)	15.4±2.9	18.9±4.3	27.2±2.2* §§	34.9±6.0**	21.4±3.6 §
C_max_ (nmol/L)	9.9±2.6	9.8±2.7	16.0±5.5* §	16.9±2.9*	10.3±3.3
t_1/2_ (h)	1.2±0.4	1.7±0.4	1.8±0.3	1.6±0.3	1.5±0.3
MRT_0–12_ (h)	1.7±0.6	2.7±0.8	2.2±0.4	2.3±0.3	2.0±0.3

* *p*<0.05 *vs* control;

** *p*<0.01 *vs* control;

§ *p*<0.05 between Rh2 5 mg/kg group and Rh2 50 mg/kg group with the same configuration;

§§ *p*<0.01 between Rh2 5 mg/kg group and Rh2 50 mg/kg group with the same configuration.

### Stereoselective LC-MS quantification of ginsenoside Rh2 epimers and the deglycosylation metabolites ginsenoside Ppd epimers

**Figure 3:**
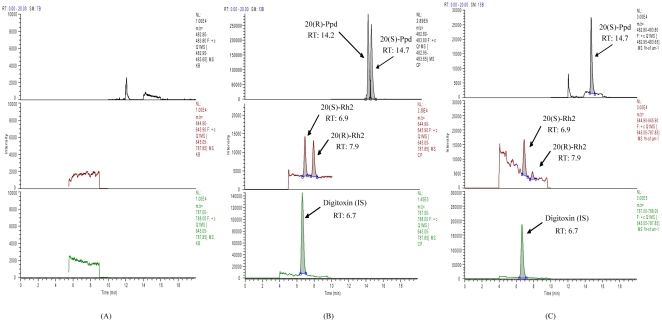
Representative SIM chromatograms of ginsenosides in biological matrix. (A) blank rat intestinal microflora suspension;(B) blank rat intestinal microflora suspension spiked with 20(S)-Rh2 (100 nM), 20(R)-Rh2 (100 nM), 20(S)-Ppd (1 µM), 20(R)-Ppd (1 µM) and digitoxin (500 nM);(C) rat intestinal microflora suspension after incubation with 1 µM 20(S)-Rh2 for 1 h.

The chromatograms shown in Fig. 3 demonstrated that the present LC-MS conditions applied for analysis of Rh2 and Ppd epimers provided appropriate separation with the retention time of 6.9, 7.9, 14.2, 14.7 and 6.7 min for 20(S)-Rh2, 20(R)-Rh2, 20(R)-Ppd, 20(S)-Ppd and digitoxin respectively. The specificity of the method was evaluated by screening blank biological matrix in selected ion monitoring (SIM) mode, and no interference had been observed. The method showed good linearity in a range of 1–1000 nM with a correlation coefficient R^2^ exceeding 0.995 for the analytes.

### Stereoselective oral pharmacokinetics of ginsenoside Rh2 epimers in rats

**Figure 4:**
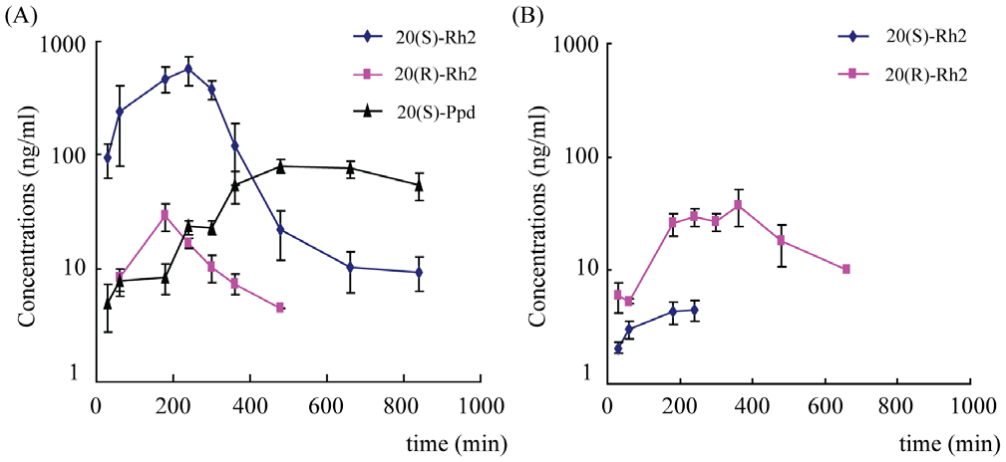
Plasma concentration-time curves of ginsenoside Rh2 epimers and their deglycosylation metabolites Ppd epimers after i.g. administration of 20(S)-Rh2 or 20(R)-Rh2 in male SD rats. (A) 25 mg/kg 20(S)-Rh2, (B) 25 mg/kg 20(R)-Rh2. Data are expressed as mean ± S.E., n = 5 per group.

As seen in Fig. 4, there was significant difference in oral pharmacokinetics of ginsenoside Rh2 epimers in rats. With the same dosage for oral administration, the C_max_ and AUC of 20(S)-Rh2 were 15-fold and 10-fold higher than those of 20(R)-Rh2 respectively: the C_max_ of 20(S)-Rh2 was nearly 1000 nM while the C_max_ of 20(R)-Rh2 was no higher than 50 nM, which suggested better oral absorption of 20(S)-Rh2 than 20(R)-Rh2 (Table 2). Furthermore, chiral inversions between ginsenoside Rh2 epimers were observed. When 20(S)-Rh2 was orally administered, 20(R)-Rh2 was also detected in plasma, with C_max_ only one eighth of 20(S)-Rh2 and AUC only one tenth of 20(S)-Rh2. Similarly, when 20(R)-Rh2 was orally administered, 20(S)-Rh2 was also detected in plasma, and the concentrations of 20(S)-Rh2 were much lower than those of 20(R)-Rh2. Otherwise, the deglycosylation metabolite of 20(S)-Rh2 was also monitored in plasma when 20(S)-Rh2 was orally administered, and the configuration of Ppd was confirmed by the standard substance of 20(S)-Ppd. But, no Ppd was found in plasma after oral administration of 20(R)-Rh2.

**Table 2:** Pharmacokinetic parameters of ginsenoside Rh2 epimers and their deglycosylation metabolites Ppd epimers after i.g. administration of 25 mg/kg 20(S)-Rh2 or 20(R)-Rh2 in rats.

	20(S)-Rh2	20(R)-Rh2	20(S)-Ppd	20(R)-Ppd	
20(S)-Rh2 25 mg/kg					
AUC _0-t_ (µg/L·min)	149888±40725	8648±1871	35129±2261	ND	
MRT _0-t_ (min)	201±15	143±27	543±21	ND	
T_max_ (min)	207±15	126±42	520±48	ND	
C_max_ (µg/L)	572±162	17±2	85±7	ND	
20(R)-Rh2 25 mg/kg					
AUC _0-t_ (µg/L·min)	1193±230	13204±2322	ND	ND	
MRT _0-t_ (min)	132±26	279±19	ND	ND	
T_max_ (min)	140±39	285±46	ND	ND	
C_max_ (µg/L)	5±1	29±5	ND	ND	

ND, Not detected.

### Stereoselective metabolic kinetics of ginsenoside Rh2 epimers by rat fecal microflora

**Figure 5:**
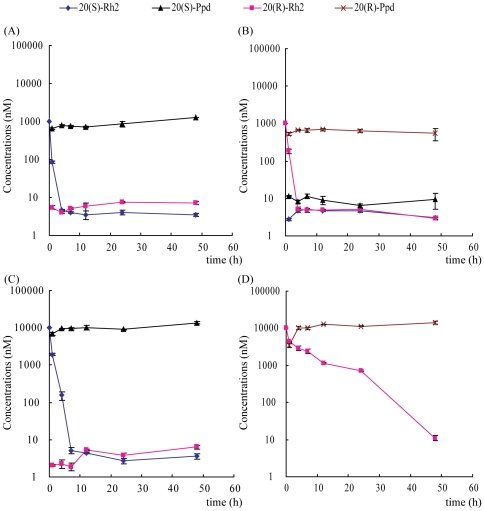
Time course of 20(S)-Rh2 and 20(R)-Rh2 transformation by rat fecal microflora *in vitro*. (A) 1 µM 20(S)-Rh2; (B) 1 µM 20(R)-Rh2; (C) 10 µM 20(S)-Rh2; (D) 10 µM 20(R)-Rh2.

Deglycosylation contributed greatly to the biotransformation of ginsenoside Rh2 with fecal microflora [18], [19]. As seen in Fig. 5A and 5C, when 20(S)-Rh2 (1 µM and 10 µM) was incubated with rat fecal microflora in anaerobic condition, the level of 20(S)-Rh2 decreased rapidly and the deglycosylation product 20(S)-Ppd appeared as soon as one hour. In addition, a very small amount of 20(R)-Rh2 was also detected throughout the incubation. However, when 20(R)-Rh2 (1 µM) was incubated with rat fecal microflora, there was a marked decrease in the level of 20(R)-Rh2, and not only a large amount of 20(R)-Ppd was found but also a small amount of 20(S)-Rh2 and 20(S)-Ppd were detected (Fig. 5B). Furthermore, when the concentrations of 20(R)-Rh2 were raised to 10 µM, the level of 20(R)-Rh2 was decreased rather slowly. In the incubation system, only 20(R)-Ppd could be detected, but not 20(S)-Rh2 or 20(S)-Ppd (Fig. 5D). The AUCs were calculated and listed in Table 3.

**Table 3:** The AUCs of ginsenoside Rh2 epimers and their deglycosylation metabolites Ppd epimers after incubation of ginsenoside 20(S)-Rh2 or 20(R)-Rh2 in rat fecal microflora.

Durg	20(S)-Rh2 (nmol/L×h)	20(R)-Rh2 (nmol/L×h)	20(S)-Ppd (nmol/L×h)	20(R)-Ppd (nmol/L×h)
20(S)-Rh2 1 µM	843.0±6.3	312.8±12.4	43363.1±2970.5	ND
20(S)-Rh2 10 µM	9491.5±404.2	206.3±11.0	492692.9±11965.2	ND
20(R)-Rh2 1 µM	202.9±8.7	1074.0±56.8	399.3±64.8	29655.9±2008.8
20(R)-Rh2 10 µM	ND	55074.3±1394.1	ND	557984.8±6457.5

ND, Not detected.

### Effects of 20(S)-Rh2, 20(R)-Rh2, 20(S)-Ppd and 20(R)-Ppd on P-gp functions in Caco-2 cells

**Figure 6:**
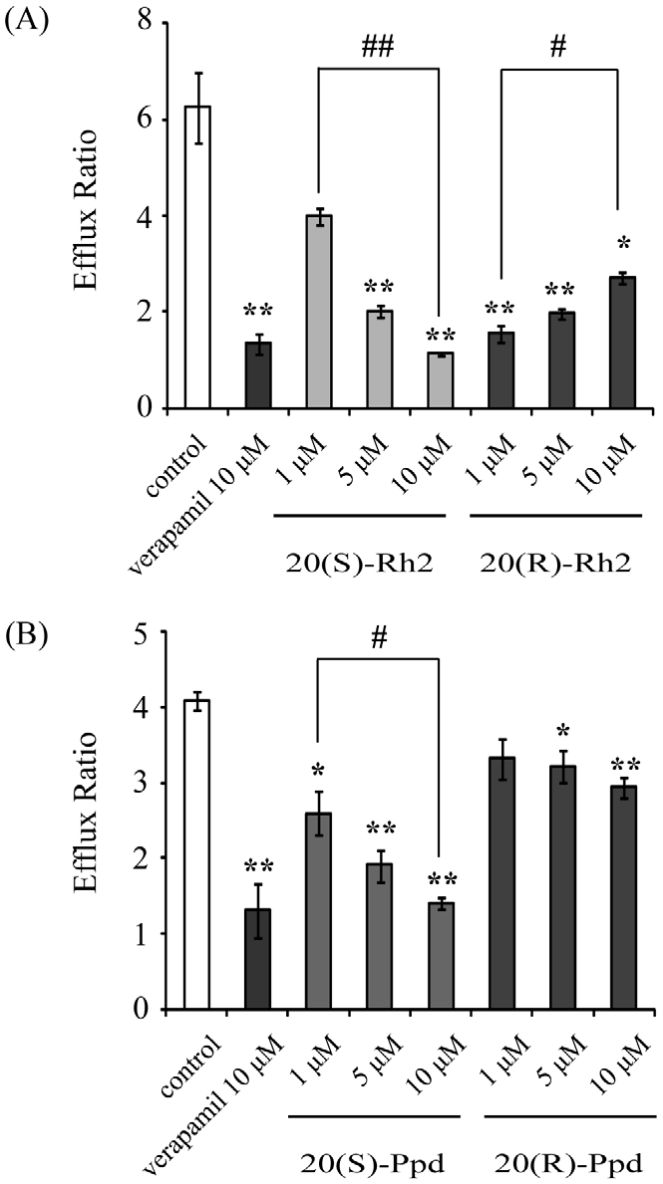
Effects of Rh2 epimers and Ppd epimers on the transport of P-gp substrate digoxin across Caco-2 monolayers. Cells were preincubated for 1 h in the presence of (A) 20(S)-Rh2 or 20(R)-Rh2, (B) 20(S)-Ppd or 20(R)-Ppd, and followed by co-incubation for 2 h in the presence of 5 µM digoxin. Data are the mean ± S.E. of three independent experiments. ^*^ *p*<0.05 *vs* control; ^**^ *p*<0.01 *vs* control; ^#^ *p*<0.05 between groups; ^##^ *p*<0.01 between groups.

Caco-2 cell model is a classic approach in the research of P-gp [20]. As shown in Fig. 6A, 20(S)-Rh2 decreased the efflux ratio of digoxin crossing Caco-2 cell monolayers in a concentration-dependent manner. However, low concentration of 20(R)-Rh2 (1 µM) significantly lowered the efflux ratio of digoxin. But, with elevated concentrations of 20(R)-Rh2, the efflux ratio of digoxin were restored.

As shown in Fig. 6B, both 20(S)-Ppd and 20(R)-Ppd lowered the efflux ratio of digoxin across Caco-2 cell monolayers concentration-dependently. But the P-gp inhibitory effect of 20(S)-Ppd was more pronounced than that of 20(R)-Ppd.

### Effects of 20(S)-Rh2 and 20(R)-Rh2 on the sensitivity of MCF-7/Adr cells to adriamycin

MCF-7/Adr cell line is an adriamycin resistant human breast cancer cell line. It is derived from the parental human breast cancer cell line MCF-7 by gradual adriamycin selection. Our previous study showed that it is more resistant to adriamycin compared with MCF-7. When series concentrations of adriamycin were added to MCF-7/Adr cells in the presence of 20(S)-Rh2 or 20(R)-Rh2, these cells exhibited differential sensitivities towards adriamycin. As seen in Table 4, 20(S)-Rh2 decreased the IC_50_ of adriamycin in MCF-7/Adr cells in a concentration-dependent manner. Although 20(R)-Rh2 (1 µM) lowered the IC_50_ of adriamycin in MCF-7/Adr cells, with increasing concentrations of 20(R)-Rh2, the sensitivity of MCF-7/Adr cells towards adriamycin was restored to the initial level concentration-dependently.

**Table 4:** The IC_50_ of adriamycin in MCF-7/Adr cells in the absence or presence of 20(S)-Rh2 or 20(R)-Rh2.

Concentrations of Rh2 (µM)	Adriamycin+20(S)-Rh2	Adriamycin+20(R)-Rh2
0	87.72±15.80	
1	89.92±17.98	57.63±7.44
5	43.20±6.91	63.34±19.01
10	0.76±0.19	80.61±15.32

## Discussion

Chirality is a basic characteristic of biological system. Investigating the stereochemistry of either biomacromolecules or exogenous small molecules plays an important role in exploring the nature of life and promoting the health of people. Especially, since the thalidomide tragedy in 1960s [21], people have realized that the racemic mixtures and individual stereoisomers could exhibit totally different physiochemical and biochemical properties including carcinogenicity and teratogenicity [22]. Developing homochiral drugs has become a demanding tendency of the pharmaceutical industry [23].

Ginsenoside Rh2 is a potential drug obtained from herbal medicines, and its stereoselective properties have also gained much attention [7], [8], [9]. In our previous studies, 20(S)-Rh2 was demonstrated as a potent P-gp inhibitor [15]. This leads us to determine whether 20(R)-Rh2 could also inhibit P-gp. We examined the effects of Rh2 epimers on the oral absorption of P-gp substrate digoxin in rats. In contrast to 20(S)-Rh2 which could promote the oral absorption of digoxin in a dose-dependent manner, 20(R)-Rh2 showed the opposite P-gp inhibitory effect.

Then, pharmacokinetic profiles of Rh2 epimers were obtained to elucidate this interesting phenomenon, assuming that different concentrations of Rh2 epimers *in vivo* might lead to differential P-gp regulations. Actually, our previous studies had shown that the stereoselectivity of Rh2 epimers was one of the factors contributing to the poor oral absorption of Rh2 [11]. However, the stereoselective absorptions of Rh2 epimers were only analyzed on models *in vitro*, without further confirmation *in vivo*. Moreover, our previous LC-MS method could not distinguish the configurations of Rh2, and therefore the potential inversions between two configurations of Rh2 were not revealed.

Hence, in our present study, a stereoselective LC-MS method for quantification of ginsenoside Rh2 epimers and the deglycosylation metabolites ginsenoside Ppd epimers were developed firstly. Then, this method was successfully applied to the stereoselective oral pharmacokinetic studies of Rh2 epimers. Although there were inversions between 20(S)-Rh2 and 20(R)-Rh2 after oral administration of a single configuration of Rh2, the inverted proportion was limited to ∼10%. This indicated that the initial configuration of Rh2 was predominant after oral absorption *in vivo*. Furthermore, 20(S)-Rh2 was absorbed into plasma more and better than 20(R)-Rh2 with higher concentrations and larger values of AUC, which was in accordance with our previous *in vitro* results [11]. Otherwise, with the fall of plasma concentrations of 20(S)-Rh2, the concentrations of the deglycosylation metabolite ginsenoside Ppd were raised, and the configuration of Ppd was approved to be 20(S)-Ppd by comparing with the authentic standard. However, 20(R)-Ppd was not detected in plasma after oral administration of 20(R)-Rh2. There are two possible reasons: one is that 20(R)-Rh2 was not deglycosylated into 20(R)-Ppd; the other is that 20(R)-Ppd was not absorbed into plasma. In order to elucidate the specific reason, metabolisms of 20(S)-Rh2 and 20(R)-Rh2 in rat fecal microflora were studied. The results showed that both 20(S)-Rh2 and 20(R)-Rh2 could be largely deglycosylated into Ppd which was the main metabolite of Rh2 in intestine [24]. The configuration of Ppd was primarily in accordance with the initial configuration of Rh2 that was added into the incubation system, which demonstrated some steps of the proposed metabolic pathway of ginsenoside Rg3 epimers by intestinal bacteria [7]. Thus, it could be concluded that 20(R)-Rh2 was deglycosylated into 20(R)-Ppd in intestine, but 20(R)-Ppd was hardly absorbed into plasma.

Through these experiments, the differential absorption of ginsenoside Rh2 epimers were confirmed *in vivo*, which indicated that the ginsenoside with R-configuration possessed lower membrane permeability and poorer absorption than the one with S-configuration. This might be attributed to the geometrical arrangement of hydroxyl groups at the chiral centers, inaccessibility to water, and compact structure of 20(S)-ginsenoside. As we all known, compounds with high hydrophobicity, weak metabolic activity and little efflux by transporters have better membrane permeability and oral absorption. For 20(S)-ginsenosides, it is speculated that hydroxyl group at carbon-20 is geometrically close to the one at carbon-12; alkene chain at carbon-20 has a stably fixed orientation and is packed tightly near the terpenoid. These characteristics prevent 20(S)-ginsenosides from accessing to water molecules. However, alkene chain at carbon-20 in 20(R)-ginsenosides protrudes further outside with flexibility, which makes 20(R)-ginsenosides more accessible to water molecule and more polar than 20(S)-ginsenosides. Therefore, more hydrophobic 20(S)-ginsenosides have better membrane permeability than 20(R)-ginsenosides [25], [26].

Furthermore, Caco-2 cells were chosen as an ideal model analyzing P-gp-mediated drug-drug interactions. The pharmacokinetic studies of Rh2 epimers *in vivo* showed that Rh2 was largely metabolized into Ppd in intestine, which suggested the unneglectable role of Ppd in regulation of P-gp. So the P-gp inhibitory effects of Rh2 and Ppd were all evaluated on Caco-2 cell monolayers using digoxin as P-gp substrate. It was found that the P-gp inhibitory effect of Rh2 epimers *in vitro* was in accordance with the studies *in vivo* from the concentration-effect viewpoint. As only a little amount of Rh2 was transformed into Ppd in Caco-2 cell incubation buffer [27], the observed differential P-gp regulation effect of Rh2 epimers could be attributed to Rh2 itself. Whereas Ppd epimers all showed inhibitory effects on P-gp function in a positive concentration-dependent manner but with different inhibitory abilities.

In view of the *in vitro* and *in vivo* dispositions of Rh2 and the regulations of P-gp by Rh2, it is indicated that the strong P-gp inhibitory effect of 20(S)-Rh2 *in vivo* actually is a double actions of both 20(S)-Rh2 and 20(S)-Ppd. However, it is complex for 20(R)-Rh2. Previously, Berginc *et al* reported that aged garlic extract increased darunavir efflux while decreased saquinavir efflux in both HepG2 cells and rat liver slices, which were attributed to different binding sites in P-gp [28], [29]. Accordingly, we put forward the following speculations. At low dosage, 20(R)-Rh2 was rapidly transformed into 20(R)-Ppd (Fig. 5B). Then, the small amount of 20(R)-Rh2 might compete with digoxin for the same binding site, and inhibited the efflux of digoxin. Otherwise, 20(R)-Ppd was largely resided in the intestine and exhibited its P-gp inhibitory effect (Fig. 6B). The net effects of 20(R)-Rh2 and 20(R)-Ppd showed inhibition. When the dosage of 20(R)-Rh2 was elevated, the transformation rate of 20(R)-Rh2 into 20(R)-Ppd was significantly lowered (Fig. 5D). Large amount of both 20(R)-Rh2 and 20(R)-Ppd were coexistent in the intestine. And 20(R)-Rh2 might not only compete for digoxin binding site, but also has affinity for other regulatory site in P-gp. This probably caused the transition of digoxin binding site from low to high affinity conformation, and resulted in higher extrusion [28]. Thus, the final net effects of 20(R)-Rh2 and 20(R)-Ppd did not exhibit remarkable P-gp regulation.

Since Rh2 epimers could differentially regulate P-gp functions *in vitro* and *in vivo*, their MDR reversal effects based on P-gp inhibition were also detected. Cell growth inhibition assay was performed on multi-drug resistant cancer cells with high P-gp expression. It turned out that 20(R)-Rh2 at low concentrations could synergistically enhance the cytotoxic effect of adriamycin. However, unlike 20(S)-Rh2, when the concentrations of 20(R)-Rh2 were increased, the synergistic effect of 20(R)-Rh2 were decreased and disappeared (Table 4), which again demonstrated the stereoselective regulation of P-gp by Rh2 epimers.

In conclusion, the differential regulations of P-gp by ginsenoside Rh2 epimers *in vivo* were observed in our present study. Considering the dispositions of Rh2 epimers themselves *in vivo* and the regulations of P-gp by Rh2 and Ppd *in vitro*, the P-gp regulatory effects *in vivo* should be a net effect of Rh2 and its deglycosylation metabolite Ppd. Upon those, the Rh2 epimers were also applied in reversal of MDR and the differential reversal effects were again observed. Our study revealed the stereoselective P-gp regulation effects of ginsenoside Rh2 epimers *in vivo* and the possible mechanisms from a view of pharmacokinetics.

## Materials and Methods

### Chemicals and reagents

20(S)-ginsenoside Rh2, 20(R)-ginsenoside Rh2, 20(S)-protopanaxadiol and 20(R)-protopanaxadiol were all purchased from Jilin University (Changchun, China). Digoxin, digitoxin and verapamil were purchased from Sigma Chemical Co. (St. Louis, MO). HPLC-grade acetonitrile and methanol were purchased from Sigma Chemical Co. (St. Louis, MO). Deionized water was prepared by Milli-Q system (Millipore, Milford, MA, USA) and was used throughout. Ethylacetate and all of other reagents, solvents were commercially available and of analytical grade.

### Animals

Male healthy Sprague–Dawley rats (200–250 g) were supplied by the Experimental Animal Breeding Center, Nanjing General Hospital of Nanjing Military Command (Nanjing, China). All the rats were maintained in room temperature (22±2°C),50–60% relative humidity and automatic day-night rhythm (12 h-cycle). The animals were acclimatized to the facilities for one week, and then fasted overnight (12 h) with free access to water prior to each experiment. Rats were randomly assigned to different experimental groups. The animal experiments in this investigation were carried out in accordance with the Guidelines for Animal Experimentation of China Pharmaceutical University (Nanjing, China) and protocol was approved by the Animal Ethics Committee of this institution.

### Effects of 20(S)-Rh2 and 20(R)-Rh2 on oral pharmacokinetics of P-gp substrate digoxin in rats

The rats were divided into five groups with five each. One group of rats received *i.g.* single dose of the vehicle (0.5% CMC-Na) serving as the control. Two groups were *i.g.* administered 20(S)-Rh2 suspended in 0.5% CMC-Na at the doses of 5 mg/kg and 50 mg/kg respectively, while another two groups *i.g.* administration of 20(R)-Rh2 suspended in 0.5% CMC-Na at the doses of 5 mg/kg and 50 mg/kg respectively. Two hours later, P-gp substrate, digoxin (0.25 mg/kg) was given to the rats by *i.g.* administration. Blood samples were collected before the P-gp substrate dosing and at 0.08, 0.17, 0.25, 0.5, 1, 2, 3, 6, 8 h post-digoxin-dosing. Plasma was obtained by centrifugation at 5000 g for 10 min and stored at −20°C before analysis. Plasma concentrations of digoxin were determined as described previously [15].

### Pharmacokinetic Studies of 20(S)-Rh2 and 20(R)-Rh2 in Rats

To investigate the differences of pharmacokinetic characteristics between 20(S)-Rh2 and 20(R)-Rh2, rats were divided into 2 groups with five rats for each group. One received a single dose of 20(S)-Rh2 intragastrically at 25 mg/kg suspended in 0.5% CMC-Na, while the other received 20(R)-Rh2 at the same dosage. Blood samples were collected at 0, 30, 60, 180, 240, 300, 360, 480, 660 and 840 min after oral administration into heparinized tubes. Plasma was obtained by centrifugation at 5000 g for 10 min and stored at −20°C before analysis.

### Metabolism of 20(S)-Rh2 and 20(R)-Rh2 in Rat Fecal Microflora

Fresh feces of healthy rats were collected and suspended in anaerobic medium (1 g ∶ 3 ml). After filtration, the rat intestinal microflora suspension was ready for anaerobic incubation of ginsenoside. An aliquot of 1 ml rat intestinal microflora suspension was spiked with 20(S)-Rh2 or 20(R)-Rh2, and then was incubated under anaerobic condition. At designated time, samples were taken for analysis.

### Cell culture

Caco-2 cells (HTB-37), obtained from American Type Culture Collection (Rockville, MD, USA), were routinely cultured in DMEM supplemented with 10% fetal bovine serum, 1% nonessential amino acids, 1 mM sodium pyruvate, and 100 U/ml penicillin and streptomycin (Gibco-Invitrogen, USA). MCF-7/Adr cells were obtained from Institute of Hematology and Blood Diseases Hospital (Tianjin, China), and cultured in RPMI 1640 supplemented with 10% fetal bovine serum, and 100 U/ml penicillin and streptomycin (Gibco-Invitrogen, USA). The cells were grown in an atmosphere of 5% CO_2_ at 37°C and cell medium were changed every other day.

### Effects of 20(S)-Rh2, 20(R)-Rh2, 20(S)-Ppd and 20(R)-Ppd on P-gp Mediated Bidirectional Transport of Digoxin across Caco-2 cell Monolayers

The Caco-2 cell transport model was established as described previously [30]. Then, Hank's balanced salt solution (HBSS) containing 20(S)-Rh2, 20(R)-Rh2, 20(S)-Ppd, 20(R)-Ppd or 0.1% DMSO (control) was loaded into both apical and basolateral chambers. After incubation at 37°C for 1 h, 5 µM digoxin was added to either apical or basolateral side to evaluate the transport in absorptive and secretory direction respectively. After incubation for just another 2 h, samples were taken from the receiving chamber for analysis. Verapamil (10 µM) was used as a positive control. Digoxin was determined by LC-MS/MS. All experiments were conducted in triplicate.

### Effects of 20(S)-Rh2 and 20(R)-Rh2 on Adriamycin Sensitivity in P-gp highly-expressed MCF-7/Adr Cells

MTT colorimetric assay was used to measure the cell growth inhibition after incubation with various concentrations of adriamycin in the absence or presence of 20(S)-Rh2 or 20(R)-Rh2 at 37°C for 72 h. The concentrations required to inhibit growth by 50% (IC_50_) were calculated from survival curves using the Bliss method.

### LC-MS analysis of 20(S)-Rh2, 20(R)-Rh2 and the deglycosylation metabolites 20(S) - Ppd and 20(R)-Ppd

The 20(S)-Rh2, 20(R)-Rh2 and the deglycosylation metabolites 20(S)-Ppd and 20(R)-Ppd were quantified simultaneously by reversed-phase LC-MS. An aliquot of 100 µl sample spiked with digitoxin as internal standard was extracted by 1 ml ethylacetate. The analysis was performed on Finnigan LC-MS system (Thermo Electron, San Jose, CA, USA) with a Lux Cellulose-1 Chiral Column (250×4.6 mm, 5 µm, Phenomenex, USA). The column and autosampler tray temperatures were 40 and 4°C, respectively. The mobile phase was consisted of methanol, acetonitrile and 0.1% formic acid with gradient elution (Table 5). Mass spectrometer was operated in positive ESI mode. MS parameters were as follows: spray voltage, 5.0 kV; sheath gas/auxiliary gas, nitrogen; sheath gas pressure, 35×10^5^ Pa; auxiliary gas pressure, 20×10^5^ Pa; ion transfer capillary temperature, 300°C. Quantification was performed using SIM mode with [M+Na]^+^ peak: m/z 645.4 for Rh2; m/z 483.3 for Ppd; m/z 787.5 for digitoxin (internal standard).

**Table 5:** The composition of the mobile phase and the gradient program.

Time (min)	Methanol (%)	Acetonitrile (%)	0.1% formic acid (%)	Flow rate (µl/min)
0	30	38	32	750
7.5	30	38	32	750
8.0		99.5	0.5	700
10.0		99.5	0.5	700
10.5	5	85	10	800
14.4	5	85	10	800
14.8	30	38	32	750
20.0	30	38	32	750

### Data analysis

The pharmacokinetic parameters of digoxin, 20(S)-Rh2 and 20(R)-Rh2 in rats were obtained by noncompartmental analysis using DAS (Drug and Statistics, version 2.1, Chinese Pharmacological Society). The area under the plasma concentration-time curve (AUC) was calculated using the trapezoidal method.

For the transport assay, the apparent permeability coefficient (P_app_) and efflux ratio (ER) were calculated as reported previously [15].

Data are expressed as mean ± S.E.. Comparisons for between-groups were performed using Student's *t* test. For multiple comparisons, one-way analysis of variance (ANOVA) followed by Post-Hoc test was adopted. The difference was considered to be statistically significant if the probability value was less than 0.05 (*p*<0.05).
